# Supplementary material for: PAX8 and MECOM are interaction partners driving ovarian cancer
Source: Nat Commun. 2021 Apr 26;12:2442. doi: 10.1038/s41467-021-22708-w (PMC8076227; doi:10.1038/s41467-021-22708-w)

Supplementary Information

PAX8 and MECOM are interaction partners driving ovarian cancer

Melusine Bleu, Fanny Mermet-Meillon, Verena Apfel, Louise Barys, Laura Holzer, Marianne Bachmann Salvy, Rui Lopes, Ines Barbosa, Cecile Delmas, Alexandra Hinniger, Suzanne Chau, Markus Kaufmann, Simon Haenni, Karolin Berneiser, Maria Wahle, Ivana Moravec, Alexandra Vissieres, Tania Poetsch, Erik Ahrné, Nathalie Carte, Johannes Voshol, Elisabeth Bechter, Jacques Hamon, Marco Meyerhofer, Dirk Erdmann, Matteo Fischer, Therese Stachyra, Felix Freuler, Sascha Gutmann, Cesar Fernandez, Tobias Schmelzle, Ulrike Naumann, Guglielmo Roma, Kate Lawrenson, Cristina Nieto-Oberhuber, Amanda Cobos-Correa, Stephane Ferretti, Dirk Schübeler, Giorgio G. Galli

Supplementary Figure 1. Identification of PAX8 interacting partners

A) Schematic representation of BioID-HA-T2A-mCherry cassette integrated at the C-terminus of the PAX8 locus by CRISPR. B) Western blot analysis of bulk cell population transfected with BioID-cassette repair template before and after mCherry FACS sorting. C) Western blot analysis of single cell clones isolated from mCherry sorted IGROV-1-PAX8-BioID-T2A-mCherry. D) Gene Ontology Biological Process enrichment analysis of proteins identified from Bio-ID MS experiment in Figure 1A. BH p-val refers to an adjusted p-value of the enrichment corrected for multiple testing according to Benjamini-Hochberg. E) List of proteins identified by BioID-MS involved in Chromatin/Transcriptional Complexes, Histones or DNA Damage. P-value refers to two-sided t-test of enrichment corrected for multiple testing. F) Ratio of expression between PRDM3 and EVI1 from TCGA dataset. Data are presented as boxplot (representing median and first and third quartiles, whiskers extend to 95th percentile), omitting outliers. Number of data points for each lineage is represented on the x-axis. Source data for western blots are provided as a Source Data file.

Supplementary Figure 2. Biochemical/Biophysical confirmation of PAX8-PRDM3 interaction

A) Western blot analyses from IVTT reactions to express LgBit tagged PAX8 truncation mutants and full length PRDM3. The picture display one representative image out of three independent experiments. B) Western blot analyses from IVTT reactions to express LgBit tagged PAX8 domains. Asterisks denote unspecific bands. The picture display one representative image out of three independent experiments. C) Representative gels from purification of the indicated recombinant proteins utilized for *in-vitro* assays. Data have been reproduced independently of recombinant protein batches produced. (D) Methyl region of 1D proton spectra recorded for different PAX8 (9–135) and PRDM3 (2–345) samples. The two top traces are the reference spectra of each protein. The third trace, PAX+PRDM3 (sum) was obtained as the mathematical sum of the two reference spectra at the top. The bottom trace, PAX+PRDM3 (experimental) was measured with an equimolar mixture of PAX8 (9–135) and PRDM3 (2–345). All spectra were recorded, processed and plotted with identical parameters. The peak labeled DSS corresponds to the internal standard DSS-d_6 used in all samples. The peaks marked with asterisks are buffer impurities. E) SDS-PAGE of recombinant PAX8 (2-328) mixed with PRDM3 (75-434) untreated or treated with DSSO. The picture display one representative image out of three independent experiments. F) MALDI-MS analysis of mixture of PAX8 and PRDM3 recombinant proteins. Note that PRDM3 (75-434) tends to dimerize even in absence of crosslinking agent. G) Peptides involved in intermolecular crosslinks from peptide mapping of CX-MS. Source data for western blots and interaction assasy are provided as a Source Data file.

Supplementary Figure 3. PAX8/MECOM silencing for functional experiments

A) Heatmap of ChIP-signal for PAX8 and PRDM3 (two replicates) centered around the union of all PAX8 or PRDM3 peaks. B) ChIP-qPCR validation for PAX8 (in red) or PRDM3 (in blue) upon PAX8 knockdown or MECOM knockdown. CTGF -8.3Kb is used as negative control region. Data are presented as mean values +/- SD from 4 biological replicates. C) Western blot analysis of NIH:OVCAR3 cells bearing shRNAs against PAX8 or MECOM used in this study. VINCULIN is used as a loading control. (*) depicts shPAX8_1581 in a different vector utilized in previous studies[9]. The picture display one representative image out of three independent experiments. D) qPCR analyses validating the efficiency of the shPAX8 (in red) or shMECOM (in blue) constructs used for RNA-seq studies in 5 ovarian cancer cell lines. Data are presented as mean values +/- SD from 3 biological replicates. E) KEGG pathway enrichment analyses for the 58 genes included in the PAX8-MECOM gene module identified in this study. P-values refer to two-sided T-test. F) Tumor volume growth (left) and qPCR validation (right) for shPAX8 (in orange shades) or shMECOM (in blue shades) in-vivo experiments for RNA-seq. Tumor growth data are presented as individual mice (n>4 per group), qPCR validation data are presented as mean values +/- SD from 4 independent mice. Source data for western blots and qPCRs are provided as a Source Data file.

Supplementary Figure 4. PAX8 and MECOM regulate a specific gene expression module.

A) Barplot showing sensitivity to PAX8 or MECOM KO as per Combined RNAi screens reported in DepMap portal. Bars are color coded by MECOM expression. B) Relative proliferation effect as measured by quantification of Colony Formation Assay experiments in multiple ovarian cancer cell lines bearing shPAX8 or shMECOM constructs. C) Western blot analysis of tumors from Figure 4C, on samples derived 1 week after treatment start. D-E) Boxplot of normalized Z-score expression of 58 genes constituting the PAX8-PRDM3 gene module identified in this study in CCLE ovarian cancer cell lines (D) or Patient derived xenografts collection (E). Cell line / PTX models are ranked by median Signature score. PAX8 and MECOM z-score expression are plotted as heatmap. Pearson Correlation (r) between PAX8 or MECOM z-score and median Signature score is reported. *** = p-value <0.001. Source data for western blots and qPCRs are provided as a Source Data file.

Supplementary Figure 1

A

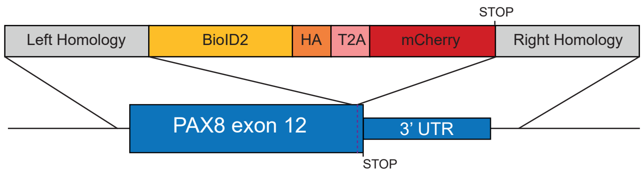

B

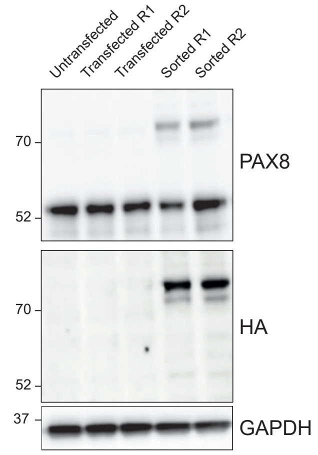

C

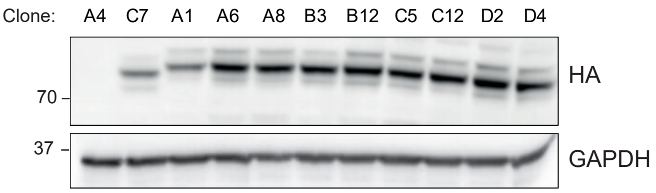

D

GO Term	Description	Fold enrich.	BH p-val
GO:0000122	Negative regulation of transcription from RNA polymerase II promoter	6.266	8.09E-07
GO:0006351	Transcription DNA-templated	3.077	1.70E-04
GO:0006281	DNA repair	9.598	6.18E-04
GO:0045944	Positive regulation of transcription from RNA polymerase II promoter	3.832	2.72E-03
GO:0045892	Negative regulation of transcription DNA-templated	5.525	2.31E-03
GO:0045893	Positive regulation of transcription DNA-templated	5.353	2.52E-03
GO:0006334	Nucleosome assembly	12.637	7.35E-03
GO:0006366	Transcription from RNA polymerase II promoter	4.886	1.05E-02

E

Chromatin / Transcriptional complexes

Protein	Accession	pval	Log2FC
INTS7	Q9NVH2	0.000479	1.816644
TET2	Q6N021	0.000659	1.658743
GATAD2B	Q8WXI9	0.000433	1.55021
LIN54	Q6MZP7	0.000557	1.540219
NCOR1	O75376	0.000476	1.524125
TLE3	Q04726	0.001525	1.446544
NCOR2	Q9Y618	0.000565	1.400802
MBD3	O95983	0.000927	1.348117
L3MBTL3	Q96JM7	0.000599	1.331513
SSRP1	Q08945	0.003234	1.326485
LRIF1	Q5T3J3	0.003694	1.317343
NCOA3	Q9Y6Q9	0.005762	1.173034
SUPT16H	Q9Y5B9	0.001579	1.156554
SMARCA4	P51532	0.002831	1.136989
SNW1	Q13573	0.0013	1.110918
TLE4	Q04727	0.007993	1.090231

Histones

Protein	Accession	pval	Log2FC
H2AFX	P16104	0.000234	2.701417
H2AFY	O75367	0.00317	2.432029
HIST1H4A	P62805	0.002282	2.405727
H1F0	P07305	0.001837	2.047347
H3F3A	P84243	0.001434	1.41105
H2AFZ	P0C0S5	0.008685	1.332943
HIST1H1A	Q02539	0.004257	1.20539

DNA Damage

Protein	Accession	pval	Log2FC
PARP1	P09874	0.000409	2.429708
TOP1	P11387	0.000773	1.929482
PARP2	Q9UGN5	0.001192	1.806807
SLX4	Q8IY92	0.000257	1.504067
LIG1	P18858	0.002169	1.298158
C11orf30	B7ZKU0	0.000752	1.148792
XPC	Q01831	0.002334	1.141888

F

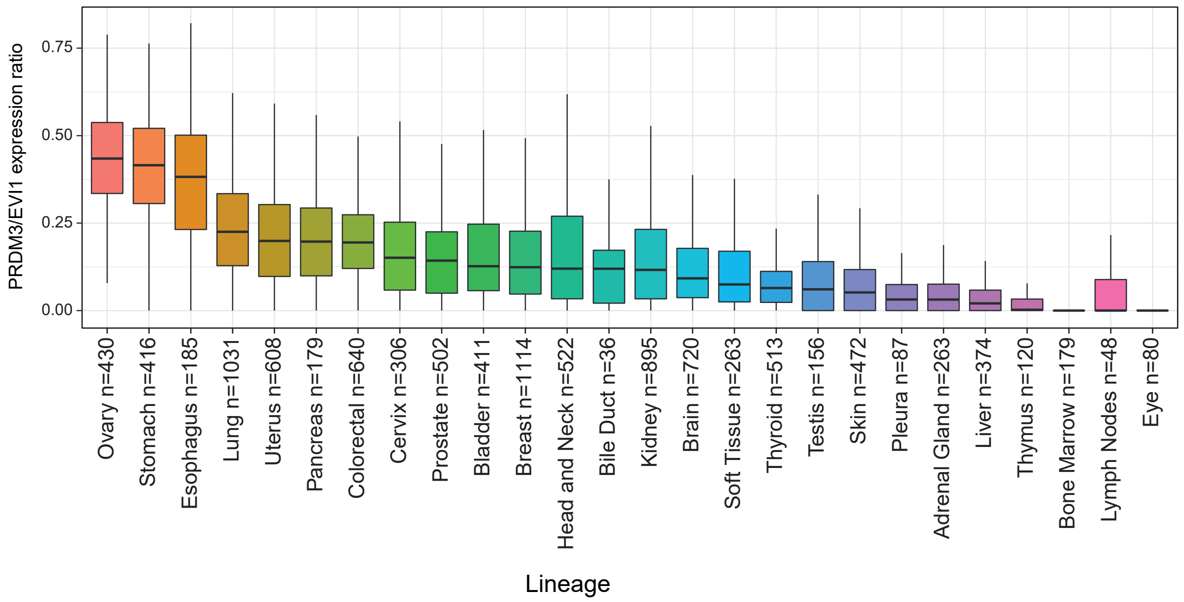

Supplementary Figure 2

A

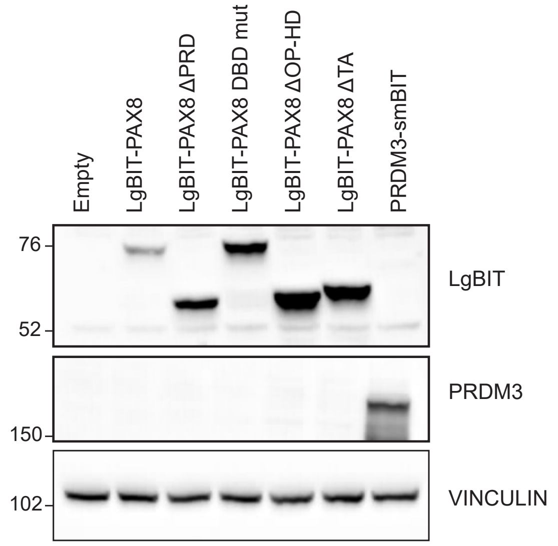

D

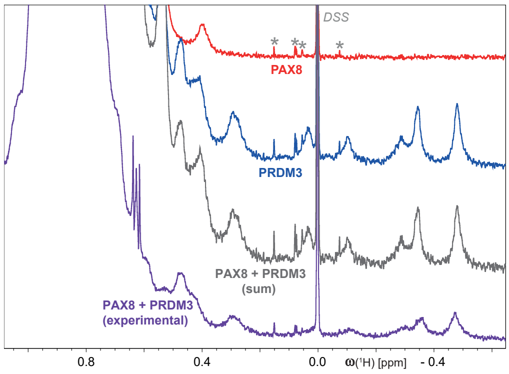

B

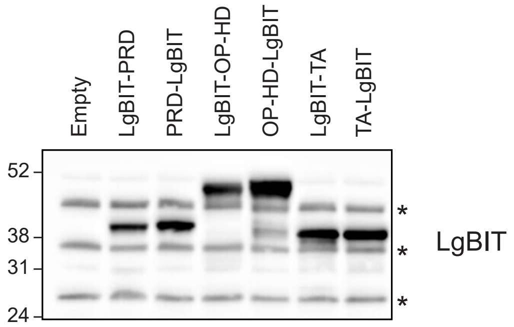

E

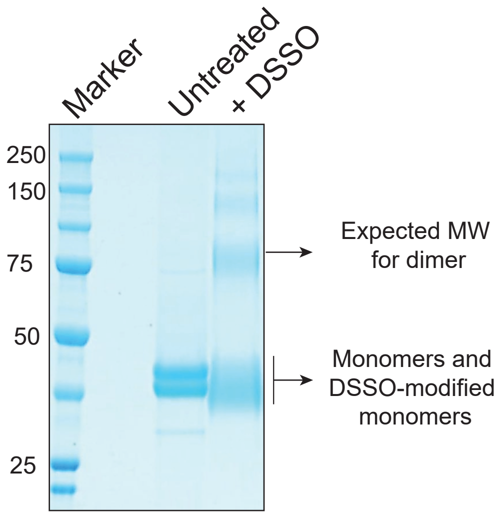

C

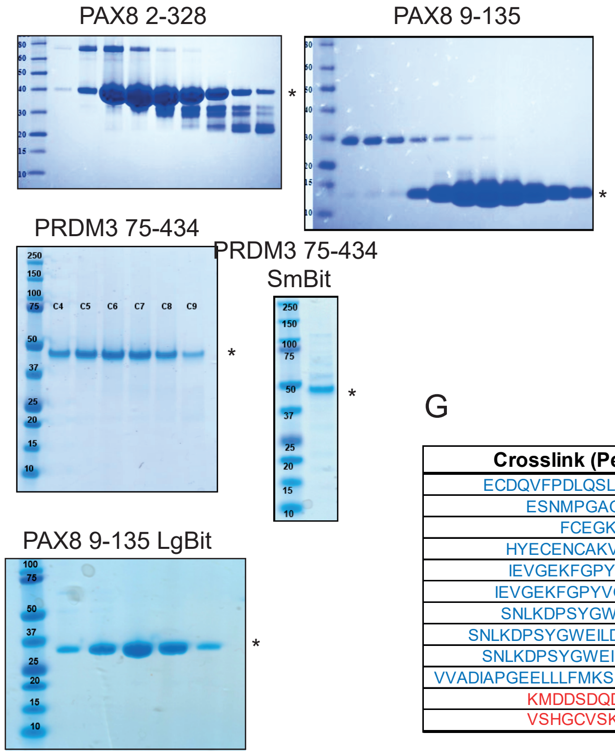

F

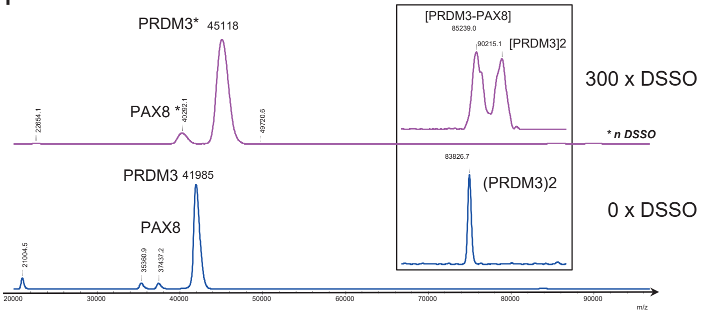

G

Crosslink (PeptideA - PeptideB)	ProteinA	ProteinB	ResidueA	ResidueB
ECDQVFPDLQSLEKHMLSHTEER-IGDYKR	PRDM3 (75-434)	PAX8 (2-328)	280	96
ESNMPGAGLGIWTKR-IGDYKR	PRDM3 (75-434)	PAX8 (2-328)	97	96
FCEGKNHFA-IGDYKR	PRDM3 (75-434)	PAX8 (2-328)	430	96
HYECENCAKVFTDPSNLQR-IGDYKR	PRDM3 (75-434)	PAX8 (2-328)	326	96
IEVGEKFGPYVGEQR-VATPKVVEK	PRDM3 (75-434)	PAX8 (2-328)	105	87
IEVGEKFGPYVGEQR-VSHGCVSKILGR	PRDM3 (75-434)	PAX8 (2-328)	105	60
SNLKDPSYGWEILDEFYNVK-IGDYKR	PRDM3 (75-434)	PAX8 (2-328)	118	96
SNLKDPSYGWEILDEFYNVK-KMDDSDQDSCR	PRDM3 (75-434)	PAX8 (2-328)	118	197
SNLKDPSYGWEILDEFYNVK-VATPKVVEK	PRDM3 (75-434)	PAX8 (2-328)	118	87
VVADIAPGEELLLFMKSEDYPHETMAPDIHEER-IGDYKR	PRDM3 (75-434)	PAX8 (2-328)	190	96
KMDDSDQDSCR-FCEGKNHFA	PAX8 (2-328)	PRDM3 (75-434)	197	430
VSHGCVSKILGR-FCEGKNHFA	PAX8 (2-328)	PRDM3 (75-434)	60	430

Supplementary Figure 3

A

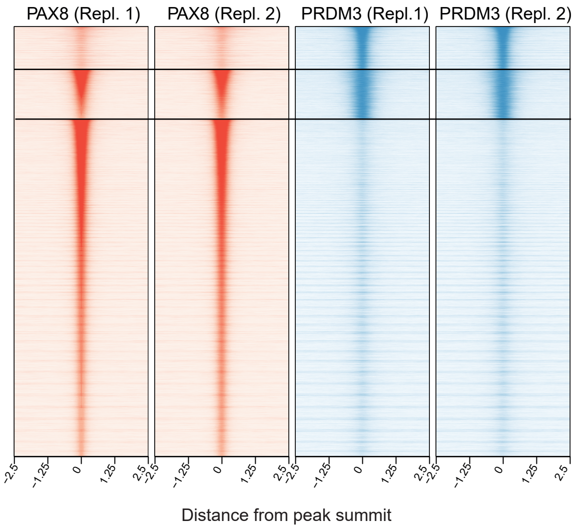

C

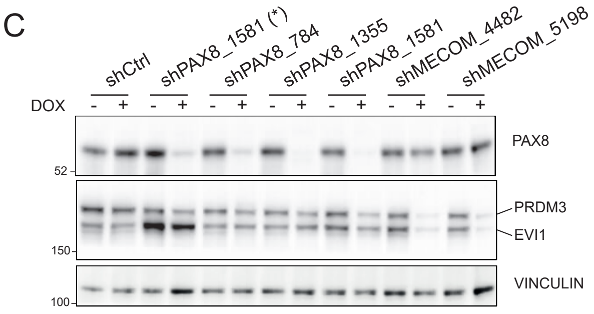

D

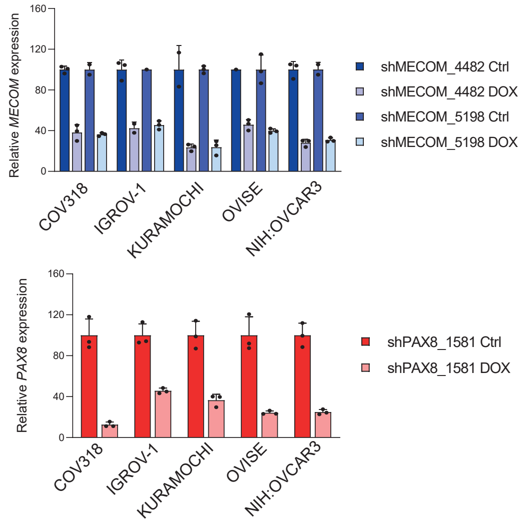

B

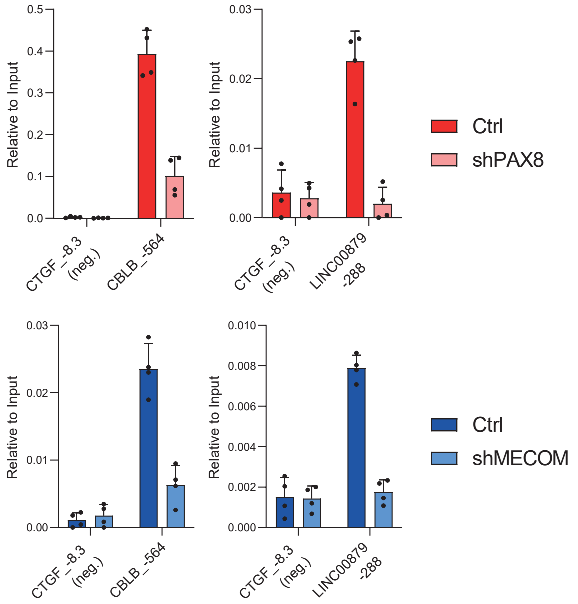

E

KEGG- Pathway Term	P-Value	Fold Enrichment	Fisher Exact
ECM-receptor interaction	3.40E-04	14.1	2.30E-05
PI3K-Akt signaling pathway	1.00E-02	4.3	2.20E-03
Focal adhesion	8.00E-03	6	1.30E-03
Regulation of actin cytoskeleton	4.80E-02	4.7	9.70E-03
TGF-beta signaling pathway	4.20E-02	8.8	4.60E-03

F

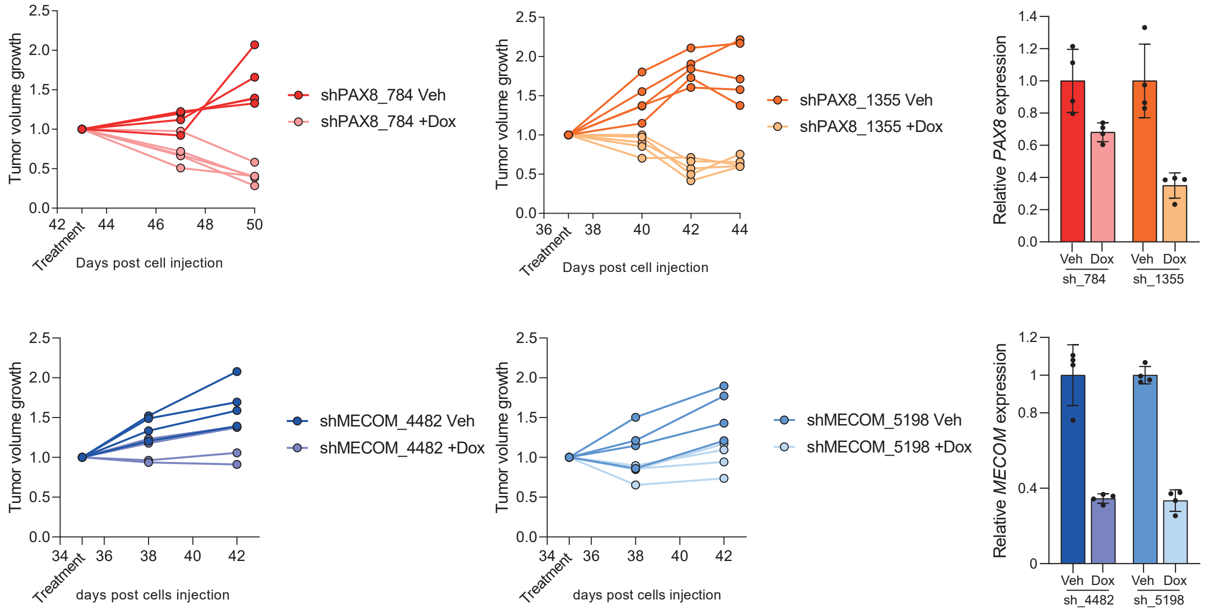

Supplementary Figure 4

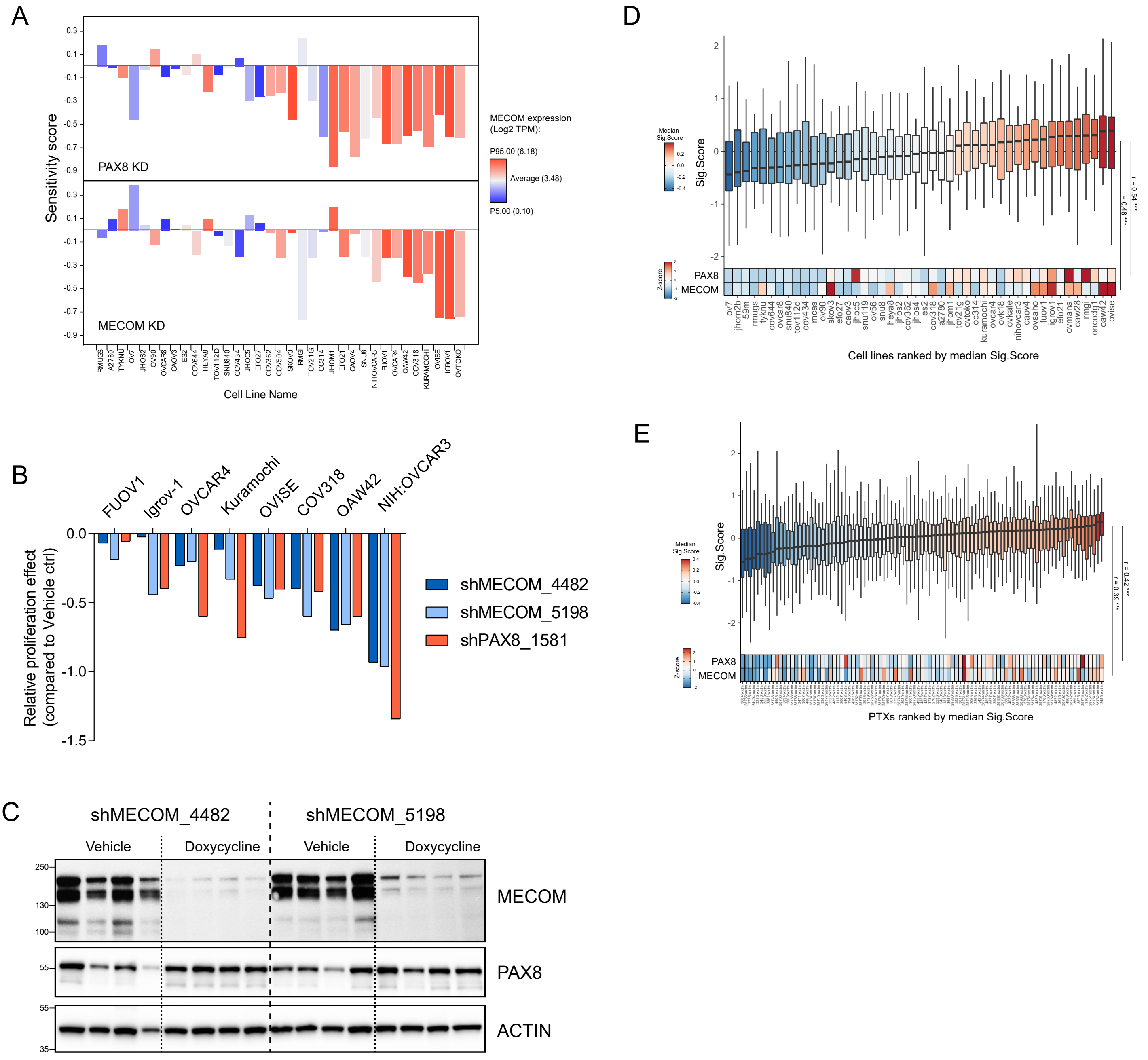

Supplement: Supplementary file 1 — Supplementary Information [file 41467_2021_22708_MOESM1_ESM.pdf]
