# Supplementary material for: PAX8 and MECOM are interaction partners driving ovarian cancer
Source: Nat Commun. 2021 Apr 26;12:2442. doi: 10.1038/s41467-021-22708-w (PMC8076227; doi:10.1038/s41467-021-22708-w)
Supplement: Supplementary file 3 — Description of Additional Supplementary Files [file 41467_2021_22708_MOESM3_ESM.pdf]

Description of Additional Supplementary Files

File Name: Supplementary Data 1
Description: Proteins identified in PAX8 BioID-MS experiment

File Name: Supplementary Data 2
Description: Inter and Intra-molecular crosslinks identified in PAX8-PRDM3 crosslinking-MS experiment

File Name: Supplementary Data 3
Description: ChIP-seq peaks for PAX8, PRDM3 and signal modulation upon reciprocal knockdown

File Name: Supplementary Data 4
Description: Full RNA-seq dataset

File Name: Supplementary Data 5
Description: SYBR green and Taqman probes used
