# Supplementary material for: PAX8 and MECOM are interaction partners driving ovarian cancer
Source: Nat Commun. 2021 Apr 26;12:2442. doi: 10.1038/s41467-021-22708-w (PMC8076227; doi:10.1038/s41467-021-22708-w)
Supplement: Supplementary file 9 — Reporting Summary [file 41467_2021_22708_MOESM9_ESM.pdf]

nature research

Corresponding author(s): Giorgio G. Galli

Last updated by author(s): Mar 8, 2021

Reporting Summary

Nature Research wishes to improve the reproducibility of the work that we publish. This form provides structure for consistency and transparency in reporting. For further information on Nature Research policies, see our Editorial Policies and the Editorial Policy Checklist.

Statistics

For all statistical analyses, confirm that the following items are present in the figure legend, table legend, main text, or Methods section.

n/a	Confirmed	
☐	☒	The exact sample size (*n*) for each experimental group/condition, given as a discrete number and unit of measurement
☐	☒	A statement on whether measurements were taken from distinct samples or whether the same sample was measured repeatedly
☐	☒	The statistical test(s) used AND whether they are one- or two-sided<br>*Only common tests should be described solely by name; describe more complex techniques in the Methods section.*
☒	☐	A description of all covariates tested
☐	☒	A description of any assumptions or corrections, such as tests of normality and adjustment for multiple comparisons
☐	☒	A full description of the statistical parameters including central tendency (e.g. means) or other basic estimates (e.g. regression coefficient) AND variation (e.g. standard deviation) or associated estimates of uncertainty (e.g. confidence intervals)
☐	☒	For null hypothesis testing, the test statistic (e.g. *F*, *t*, *r*) with confidence intervals, effect sizes, degrees of freedom and *P* value noted<br>*Give P values as exact values whenever suitable.*
☒	☐	For Bayesian analysis, information on the choice of priors and Markov chain Monte Carlo settings
☒	☐	For hierarchical and complex designs, identification of the appropriate level for tests and full reporting of outcomes
☐	☒	Estimates of effect sizes (e.g. Cohen's *d*, Pearson's *r*), indicating how they were calculated

Our web collection on statistics for biologists contains articles on many of the points above.

Software and code

Policy information about availability of computer code

Data collection	Standard software from manufacturers (Illumina Inc. and Thermo Scientific) for NGS-based and Mass Spectrometry-based data collection has been used (Proteome Discoverer version 2.1)
Data analysis	Statistics were calculated by Microsoft Excel 2016, GraphPad Prism v.8 and standard Bioconductor R (v 4.0.3) packages.<br>For NGS and MS data analysis, open source code have been used and listed in the material and methods. No proprietary code/softwares have been employed.

For manuscripts utilizing custom algorithms or software that are central to the research but not yet described in published literature, software must be made available to editors and reviewers. We strongly encourage code deposition in a community repository (e.g. GitHub). See the Nature Research guidelines for submitting code & software for further information.

Data

Policy information about availability of data

All manuscripts must include a data availability statement. This statement should provide the following information, where applicable:

- Accession codes, unique identifiers, or web links for publicly available datasets
- A list of figures that have associated raw data
- A description of any restrictions on data availability

The Crosslinking_MS data used in this study are available in the PRIDE database under accession code PXD021708 (https://www.ebi.ac.uk/pride/archive/projects/ PXD021708). The BioID-MS data generated in this study have been deposited in the PRIDE database under accession code PXD021709 (https://www.ebi.ac.uk/ pride/archive/projects/ PXD021709). ChIP-seq and RNA-seq data have been deposited in SRA under accession PRJNA655844 (https://www.ncbi.nlm.nih.gov/sra/?term=PRJNA655844) and PRJNA655836 (https://www.ncbi.nlm.nih.gov/sra/?term=PRJNA655836) respectively. The remaining data are available within the Article, Supplementary Information or available from the authors upon request.

Field-specific reporting

Please select the one below that is the best fit for your research. If you are not sure, read the appropriate sections before making your selection.

☒ Life sciences ☐ Behavioural & social sciences ☐ Ecological, evolutionary & environmental sciences

For a reference copy of the document with all sections, see nature.com/documents/nr-reporting-summary-flat.pdf

Life sciences study design

All studies must disclose on these points even when the disclosure is negative.

Sample size	Experiments were performed using sample sizes based on standard protocols in the field. No statistical test was performed to predetermine sample size but to ensure the possibility of performing statistical tests at least 3 biological replicates were performed whenever possible. Experiments involving mouse xenografts, due to intrinsic higher variability were performed with at least 4 independent mice per group.
Data exclusions	No data were excluded.
Replication	All experiments were repeated at least for three times. Detailed information on replicates was available in the figure legends. All attempts to replicate the experiments performed here were successful.
Randomization	Samples were processed in a randomized fashion.
Blinding	Data acquisition in the studies, including animal experiments, was conducted in a blinded manner. Briefly, large cohorts of mice were injected with the indicated cell lines and at a given timepoints groups were randomized to retain equal distribution of tumor volumes.<br>For the other experiments, samples were all processed in parallel in a blind fashion by assigning numerical IDs to the samples so that the operator would not have pre-knowledge of the nature of the samples.

Reporting for specific materials, systems and methods

We require information from authors about some types of materials, experimental systems and methods used in many studies. Here, indicate whether each material, system or method listed is relevant to your study. If you are not sure if a list item applies to your research, read the appropriate section before selecting a response.

Materials & experimental systems

n/a	Involved in the study	
☐	☒	Antibodies
☐	☒	Eukaryotic cell lines
☒	☐	Palaeontology and archaeology
☒	☐	Animals and other organisms
☒	☐	Human research participants
☒	☐	Clinical data
☒	☐	Dual use research of concern

Methods

n/a	Involved in the study	
☐	☒	ChIP-seq
☒	☐	Flow cytometry
☒	☐	MRI-based neuroimaging

Antibodies

Antibodies used	PAX8 CST 59019, PRDM3 (custom-made at Genscript using recombinant PRDM3 1-200 as antigen), Vinculin Sigma V9131, NanoLuc R&D systems MAB10026, EVI-1 CST 2593, Actin MAB1501, GAPDH CST 3683. For western blots, antibodies were used at dilutions stated in source data file. For ChIP-seq, PAX8 antibody was used at 1:10 dilution while PRDM3 antibody was used at 1.5ug/ChIP reaction.
Validation	Antibodies were validated by RNAi targeting experiments or cell lines displaying differential expression as reported in figure S3C.

Eukaryotic cell lines

Policy information about cell lines

Cell line source(s)	ATCC:<br>- NIH:OVCAR3<br>- COV318<br>- IGROV-1<br>- KURAMOCHI<br>- OVISE<br>- 293T

	- OV56<br>- NCI-H1299
Authentication	Cell line identity was confirmed by regular SNP array genotyping
Mycoplasma contamination	Cell lines were regularly confirmed to be negative for mycoplasma contamintation by PCR testing.
Commonly misidentified lines<br>(See ICLAC register)	No commonly misidentified cell lines were used

ChIP-seq

Data deposition

☒ Confirm that both raw and final processed data have been deposited in a public database such as GEO.

☒ Confirm that you have deposited or provided access to graph files (e.g. BED files) for the called peaks.

Data access links<br>*May remain private before publication.*	Search in SRA https://www.ncbi.nlm.nih.gov/sra<br>- SRP276625 and BioProject PRJNA655836<br>- SRP276627 and BioProject PRJNA655844<br>Full list of peaks (BED file) are provided in Supplementary Data 3
Files in database submission	FastQ
Genome browser session<br>(e.g. UCSC)	N/A. Data from repository are freely accessible to generate BigWig files.

Methodology

Replicates	Duplicates
Sequencing depth	>30 milion uniquely mapped reads
Antibodies	IgG Sigma I506 (1ug), PAX8 CST 59019 (1:10), Custom PRDM3 antibody (1.5 ug/ChIP), H3K27ac CST 8173 (1:20), H3K4me3 Millipore 07-473 (1ug), H3K27me3 CST 9733 (1:20)
Peak calling parameters	MACS2 mfold 5, fdr 10-E09
Data quality	FRIP score was calculated. For PAX8 >20000 peaks and for PRDM3 >6000 were identified with peak calling parameters described
Software	Standard software packages were employed.
